# Clinical relevance of interdental papilla biopsy in chronic erosive gingivitis (desquamative gingivitis): retrospective bicentric study of 148 specimens

**DOI:** 10.1186/s12903-021-01820-9

**Published:** 2021-09-17

**Authors:** Frédérick Gaultier, Anne-laure Ejeil, Sébastien Jungo, Saskia Ingen-Housz-Oro, François Le Pelletier de Clatigny, Gogly Bruno, Philippe Pirnay, Fadel Bellakhdar, Sophie-Myriam Dridi

**Affiliations:** 1 Department of Odontology, Oral Medicine and Oral Surgery, APHP Henri Mondor Hospital, Université de Paris, 1 Rue Gustave Eiffel, 94000 Créteil, France; 2 Department of Odontology, Oral Medicine and Oral Surgery, Bretonneau Hospital, Université de Paris France, Paris, France; 3 Department of Dermatology, Henri Mondor Hospital, Créteil, France; 4 Competence Centre of Autoimmune Bullous Diseases MALIBUL, FIMARAD Sector, Créteil, France; 5 EA7379 EpidermE, UPEC, Créteil, France; 6 Department of Pathology, Pitié Salpetrière Hospital, Paris, France; 7 Laboratory of Molecular Oral Pathophysiology, INSERM 1138, Université de Paris France, Paris, France; 8 Department of Odontology, Saint Roch Hospital, Nice, France; 9 Oral Microbiology, Immunotherapy and Health EA 7354, Nice, France

**Keywords:** Biopsy, Erosive gingivitis, Desquamative gingivitis, Oral lichen planus, Autoimmune bullous diseases

## Abstract

**Background:**

Chronic erosive gingivitis, also called desquamative gingivitis, defines a clinical picture that can be generated by several inflammatory and immune diseases. Pathology is therefore essential for the differential diagnosis. However, when the gingival lesion is initial, exclusive or predominant, selecting the biopsy site and protocol may be problematic due to tissue fragility. Especially since there are few studies on the subject, the aim of our study was to assess the protocol, diagnostic relevance and tolerance of an original protocol using interdental papilla biopsy.

**Methods:**

We conducted a retrospective bicentric study, from October 2011 to July 2019, including all patients with a chronic erosive gingivitis who had received, for diagnostic purposes, a interdental papilla biopsy.

**Results:**

The contribution levels for the two hospital departments were 94.7% and 97.1%, respectively. No postoperative complication was recorded in the short or long term.

**Conclusion:**

The interdental papilla biopsy protocol is perfectly adapted to the anatomopathological examinations required to establish differential diagnosis of chronic erosive gingivitis. This surgical protocol is simple to perform, non iatrogenic with a very good tolerance and and accessible to all clinicians. It is highly efficient with an excellent contribution level.

*ClinicalTrials* NCT04293718 (March 3, 2020). Health Data Hub N° F20201109083211 (November 9, 2020).

**Supplementary Information:**

The online version contains supplementary material available at 10.1186/s12903-021-01820-9.

## Background

Chronic erosive gingivitis defines a clinical picture associating a pronounced gingival inflammation with gingival erosions. In the literature, the term of «desquamative gingivitis» is also often used to define it. However, this definition is inappropriate because the physiopathological process that leads to this gingival disease does not induce desquamation but a loss of tissue, i.e. erosion, involving all or part of the oral gingival epithelium. Indeed, desquamation of the buccal gingival epithelium results in the spontaneous elimination of the keratinized layer which is counterbalanced by cell divisions within the basal lamina, thus avoiding altering tissue continuity. This type of gingivitis evolves in phases of variable duration. Gingivitis is readily diffuse, haemorrhagic, and more or less generalized. It can involve the total height of the gingiva. The presence of pseudomembranes corresponding to epithelial necrotic debris is also possible, less frequently blisters with a clear, cloudy-like or hemorrhagic content, which indicate an epithelial detachment. Gingival pain is constant, which greatly reduces the efficiency of oral hygiene. Moreover, when gingival erosions are severe and extensive, feeding is difficult, and patients’ quality of life is significantly altered. Lastly, non-resolution of the gingival inflammation following periodontal treatment is often described both by the clinician and the patients. In most clinical situations, this non plaque induced gingivitis is an oral manifestation of inflammatory and immune diseases, classified in the category inflammatory and immune conditions and lesions of the new classification of the periodontal diseases [1, 2].

In most clinical situations, this syndrome is an oral manifestation of a general dysimmune disease. The most commonly described diseases are gingival lichen planus and autoimmune bullous diseases [3, 4].

Lichen planus, essentially idiopathic, is a T lymphocyte-mediated mucocutaneous disease. Oral lichen planus is the most common mucous form. When it is erosive and develops over several years, the risk of transforming into squamous cell carcinoma is increased even within the gingiva [5–7]. Autoimmune bullous diseases, less known to dental clinicians, are a heterogeneous group of rare diseases, with variable prognosis, and sometimes severe [8, 9]. These acquired diseases are mediated by autoantibodies to keratinocytic junction systems, and are characterized by the formation of intra-epithelial or sub-epithelial blisters which alter the structure and function of squamous epithelia. Among these diseases, some have a mucocutaneous expression and involve the oral mucosal membranes, particularly the gingiva, such as pemphigus vulgaris, mucous membrane pemphigoid (or cicatricial pemphigoid), acquired bullous epidermolysis, linear immunoglobulin A dermatosis and pemphigoid lichen planus [10]. The last four disorders are grouped into the category of autoimmune subepithelial bullous diseases, as opposed to pemphigus diseases which generate intraepithelial blisters. Besides these diseases, plasma cell gingivitis is an uncommon inflammatory condition, rare and benign, that is clinically similar to chronic erosive gingivitis. For some authors, this disease would be caused by a type IV hypersensitivity reaction to a natural or synthetic environmental allergen, following its repeated contact with the gingiva or its penetration within the gingival epithelial layers [11, 12].

The differential diagnosis requires a complete semiological analysis and an additional anatomopathological examination: standard histology examination and direct immunofluorescence. The quality of the oral mucosa biopsies is therefore a prerequisite sought by all clinicians.

The biopsy site must be accessible and representative of the lesion. The surgical procedure must be the least iatrogenic and the most reproducible as possible, in order to avoid repeated biopsies, feared by patients.

Several techniques for oral mucosa biopsies are currently proposed, but few of them have been methodically described.

In 2018, as part of a retrospective monocentric study, we were the first to detail an original protocol for interdental ginigival biopsy, which was non iatrogenic, perfectly adapted for the required anatomopathological diagnosis in autoimmune bullous diseases with gingival expression [13].

We propose a second retrospective, two-center study to assess the clinical relevance of that protocol, by including this time the differential diagnoses of the chronic erosive gingitis condition.

The clinical impact is real because in many countries, including France, the distribution of clinicians specialized in oral mucosa pathologies is variable, their number is insufficient, and the waiting time to get an appointment may be particularly long. The involvement of general clinicians in detecting diseases that can cause an chronic erosive gingivitis condition is therefore necessary to reduce diagnostic delays and refer the patient early to a dermatology referring hospital in case of autoimmune disease diagnostic.

## Methods

### Study population and study design

We analysed data derived from a retrospective two-center diagnostic study which was carried out from October 2011 to July 2019, in two departments of Oral Medicine with two hospitalo-university groups: Bretonneau in Paris and Henri-Mondor in Créteil (France). These two departments are specialized in the management of oral mucosa pathologies (ClinicalTrials.gov identifier NCT04293718, Health Data Hub N° F20201109083211).

All the patients were referred for outpatient consultation by their regular doctor or dental surgeon. They presented an erosive gingivitis in acute phase, isolated or predominant compared to other oral lesions requiring at least one biopsy for diagnosis purposes. Patients were included in the study, regardless of their age or general health condition. For each of them, an intraoral clinical examination was performed before implementing the gingival biopsies. The exclusion criteria were the following: patients referred with a histological examination and immunofluorescence (DIF) previously performed, and patient under corticosteroid therapy resulting in biased DIF data.

### Selection and processing of tissue specimens

The patients were informed of the potential therapeutic risks related to the tissue specimens, and had all given their informed consent. Gingival biopsies were performed by senior dental clinicians, residents or postgraduate students in Oral Surgery or Periodontology. Two simultaneous biopsies, one essential for a standard histological examination, and the other for direct immunofluorescence, are recommended in case of atypical, severe, long-standing chronic erosive gingivitis or chronic erosive gingivitis associated with blisters or pseudomembranes. For the moderate clinical forms, only one biopsy is performed for histological examination. If the results only confirm an erosive lichen planus, the second biopsy for DIF is not performed, because it would be useless (DIF necessarily negative). Therefore, all the biopsies were obtained in a similar manner. The gingival biopsy sites was selected according to the severity of gingival inflammation. We followed the usual recommendations by performing the interdental papilla biopsy distant from gingival erosion or particularly inflammed gingival regions [14, 15]. Indeed, in these clinical situations, the risk of tissue damage is maximum at the time of biopsy. Furthermore, in case of autoimmune bullous diseases, the autoantibodies are destroyed by the inflammatory response which is responsible for the epithelial cleavage [16, 17].

**Fig. 1:**
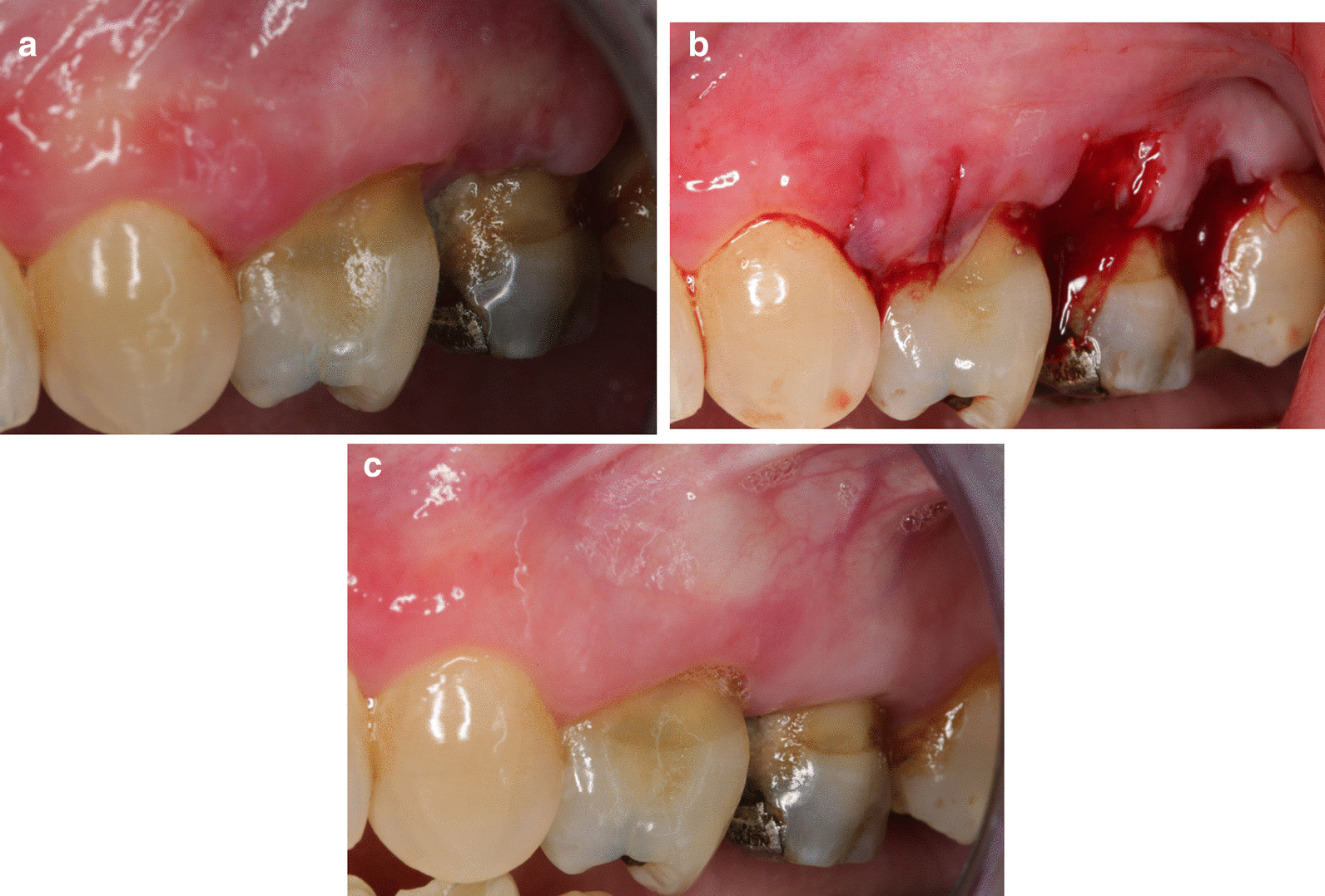
Interdental papilla biopsies performed on a female patient with mucous membrane pemphigoid (**a**). On the first selected papilla (1), the epithelium cleaved immediately after the primary incisions. Incision of this papilla was therefore stopped. The biopsy was reported to the next adjacent papillae (2 and 3) (**b**). At 21 days following surgery, healing of the 3 papillae is complete (**c**)
- Dissection of the gingival specimen after tilting the blade parallel to the interdental cortical bone, always keeping bone contact.
- Collecting the gingival specimen on the back of the blade, without using a tissue tweezer which could crush the biopsy.
- Compression of the operating site using sterile compresses. No suture is required for hemostasis. In case of an obvious hemorrhagic risk (patient under anticoagulant), surgical glue or nitrocellulose pad was applied on the surgical site.

Our surgical protocol included the following steps (movie, online resource 1):

- Elimination of the supragingival plaque using a compress soaked with povidone iodine (Bétadine® 10% for oral use) or 0,12% chlorhexidine in case of iodine allergy, in order to limit the risk of intraoperative bacteremia and toxemia.
- Periapical anesthesia, with vasoconstrictor, without intrapapillary injection to avoid blister formation and disruption of the gingival connective tissue (half a cartridge of articaine hydrochloride 1/200000).
- Sharp and clear-cut intrasulcular incisions following the coronal contours of the buccal papilla, then extending beyond its base perpendicular to the epithelial surface, up to bone contact, without ever reaching the mucogingival junction. Incisions were performed using a conventional n°15 or 15C blade. Blood was absorbed via sterile compresses instead of surgical suction to preserve the gingival specimen. In case of visible epithelium cleavage (white membrane detachment), biopsy was interrupted and carried over to another papilla of the anesthetized region (Fig. 1a, b).

At the end of surgery, patients received the usual recommendations for postoperative hemostasis procedures. A local antiseptic gel or mouthwash, containing 0.12% chlorhexidine, was prescribed 2–3 times per day, starting on the day after the biopsy, during for 3 days or less, and a level I pain reliever (Paracetamol 1 g) as well. A control visit was systematically scheduled between day 10 and day 21 following surgery during which patients were informed of the results. More than half of them were able to benefit from periodontal follow-up during several months implemented by the senior dental clinicians.

At least one biopsy per patient was placed in formaldehyde, for standard HE pathological analysis; a second biopsy, required for direct immunofluorescence, was performed either immediately following the first one, or in a second step, after reading the first standard HE pathological report; this second biopsy is placed in Michel’s solution.

### Apropriate transportation/fixative liquid depends on the question the clinician asks to the pathologist

Formaldehyde is for the standard hematoxylin and eosin staining allows the pathological report allows several diagnosis. Michel’s solution is used the direct immunofluorescence, allowing a more specific pathological report. Usually both are convenient with an appropriate description of the clinical aspect. In both transportation / fixative liquid the biopsy can stay as long as many weeks at room temperature.

### Diagnostic criteria and diagnosis

The definite diagnosis for disease causing the chronic erosive gingivitis condition were established comparing clinical data to pathological criteria commonly accepted by the international community (Table 1) [2, 3, 6, 18, 19]. Patients who required medical management were adressed to the referring doctors of both hospitals.

**Table 1:** : Main etiologies that can generate chronic erosive gingivitis also called desquamative gingivitis; clinical, histological and immunohistological characteristics

	Main clinical characteristics	Standard pathological examination (HE) Direct immunofluorescence (DIF)
*Erosive GLP*		
Typical	Erosive gingivitis usually bilateral affecting oral mucosa and/or tongue (reticular patches with or without erosions), no blisters No indirect Nikolsky’s sign	HE: basal cell degeneration, necrosis of basal and parabasal keratinocytes, predominant band-like lymphocytic infiltrate adjacent to basal cells, inflammation may include plasma cells DIF−: absence of linear deposits of IgG, IA and C3 along the epithelial basement membrane, but sometimes nonspecific marking of colloid bodies HE: similar to GLP, but the inflammatory infiltrate, may extend within the deep lamina propria DIF−: similar to GLP HE: similar to GLP with epithelial cleavage DIF−: similar to GLP
Related (oral lichenoid lesions)	Either similar to typical GLP or more or less erosive	
Bullous	Particularly erosive GLP, presence of blisters but no oral indirect Nikolsky’s sign	
*AIBD*		
MMP (CP)	Erosive gingivitis: well limited erosions, greyish yellow fibrinous background, and surrounded by a more or less extensive inflammatory halo, with no reticular component; rare intact blisters, ± other oral mucosa sites affected (oral mucosa and palate) Possible erosive zones covered with pseudomembranous, suggestive sign if associated with oral indirect Nikolsky’s’ sign	HE: subepithelial cleavage with no acantholysis, inflammatory infiltrate of neutrophils and eosinophils DIF+: Linear deposit of IgG, and C3 along the epithelial basement membrane, often associated with IgA
PLP	Erosive gingivitis: combined characteristics of lichen planus and mucous membrane pemphigoid (possible oral indirect Nikolsky’s sign)	HE: characteristics of GLP or MMP or both DIF+: similar to MMP
PV	Erosions with jagged edges and a dull-red background, circled with white areas of leukemia, no oral indirect Nikolsky’s sign	HE: acantholysis with supra-basal intraepithelial cleavage DIF+: IgG and C3 deposits on the surface of the keratinocytes (« fishnet» or « honeycomb» feature)
*PG*	Erosive gingivitis similar to GLP	HE: Spongiosis within the epithelium, dense bunched inflammatory infiltrate areas, mainly composed of plasma cells associated with a few polymorphs DIF−

*GLP* gingival lichen planus, *AIBD* autoimmune bullous diseases, *MMP* mucous membrane pemphigoid, *CP* cicatrical pemphigoid, *PLP* pemphigoid lichen planus, *PV* Pemphigus vulgaris, *PG* plasma cell gingivitis

### Outcome measures

*Primary outcome measure* To assess the efficiency of the papilla biopsy technique, we considered all the situations for which biopsies were not contributory in first intention to establish a definite diagnosis, and required an additional biopsy.

*Secondary outcome measure* To assess the tolerance of our surgical protocol, we also recorded the potential postoperative complications:

- immediately after surgery: persistent bleeding
- 24 h after surgery: persistent bleeding
- 1 week after surgery: ecchymosis, oedema, and pain assessed using a digital visual analog scale pain, scoring the pain from 0 to 10
- 14 days after surgery: delayed wound healing, incomplete interdental papilla regeneration
- 3 months or more after surgery: formation of gingival fibrotic scars, loss of the interdental papilla.

## Results

### Retrospective study

Over the study period, 101 files were reviewed. However, our final patient sample included 100 adults (mean age: 61.4 years; 72 females, 28 males), because we decided to exclude a 13 y.o adolescent, in order to avoid biased results. First, 2 biopsies of interdental papilla were simultaneously performed on 48 patients (48%), 1 for histological examination and 1 for immunohistochemistry. For 47 other patients (47%), only 1 biopsy was performed for histological examination, and for the remaining 5 patients (5%), only 1 biopsy for direct immunofluorescence because histological examination had already been prescribed by the dental clinician or by the referring doctor.

A total of 148 interdental papilla biopsies were performed by several clinicians selected among our two staffs (oral surgeons or senior periodontologists, residents or postgraduate students): 95 for histological examination and 53 for direct immunofluorescence (Additional file 1).

Only 5 biopsies (3.4%) were not contributory to establish the exact diagnosis. This involved 4 patients with mucous membrane pemphigoid, 2 for each hospital. The reason given by the anatomopathologists was the absence of epithelium in the gingival specimen; 1 biopsy was for standard histological examination, and 4 for direct immunofluorescence (Table 2). Three biopsies had been performed in 2 patients medicated with drugs altering hemostasis, and in all cases, erosive gingivitis was generalized, old-standing, and particularly severe. Additional biopsies were performed by the same clinicians and revealed contributory.

**Table 2:** **:** Number of firstline biopsies performed, for standard pathological examination (HE) or direct immunofluorescence, depending on the type of pathology and hospital department

	Contributive biopsies	Non contributive biopsies (absence of epithelium)	Total of biopsies
*GLP*			
Bretonneau Hospital	17 HE, 4 IFD	0	21
Henri Mondor Hospital	46 HE, 16 IFD	0	62
*AIBD*			
Bretonneau Hospital	8 HE, 6 IFD	2 IFD	16
Henri Mondor Hospital	19 HE, 23 IFD	1 HE, 2 IFD	45
*PG*			
Henri Mondor Hospital	3 HE	0	3
*PIG*			
Bretonneau Hospital	1 HE	0	1
Total of biopsies	143	5	148

*GLP* gingival lichen planus and related, *AIBD* autoimmune bullous diseases, *PG* plasma cell gingivitis, *PIG* plaque induced gingivitis

**Fig. 2:**
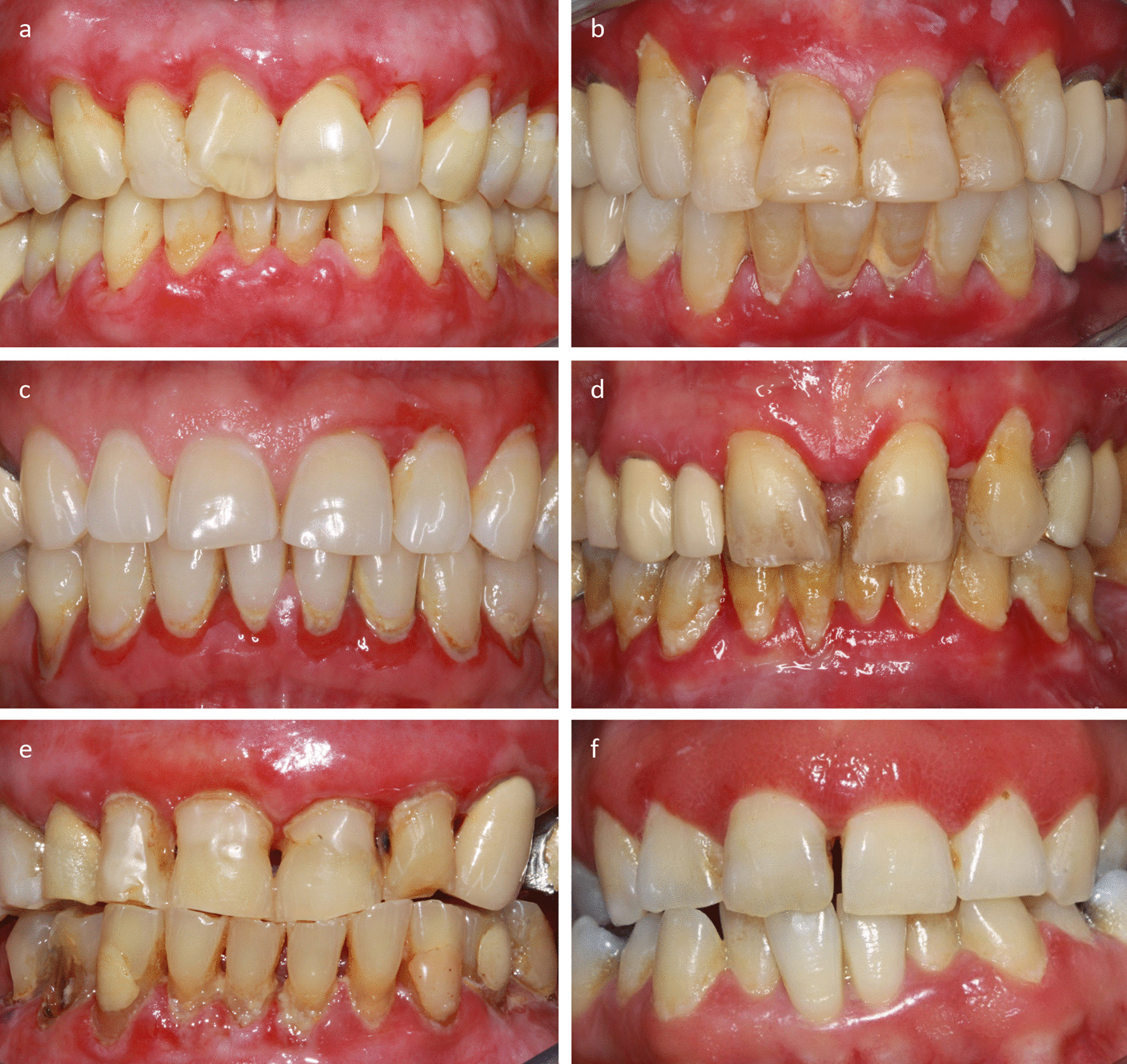
Clinical examples of systemic inflammatory and autoimmune diseases with predominantly gingival expression, diagnosed with the papillary biopsy technique. In all these patients, the gingiva was particularly inflammatory, hemorrhagic at the slightest touch and erosive in several places (clinical picture of desquamative gingivitis). The diagnostic delay varied from a few months to several years. **a** Gingival lichen planus; **b** bullous gingival lichen planus, **c** pemphigus vulgaris; **d** mucous membrane pemphigoid; **e** pemphigoid lichen planus; **f** plasma cell gingivitis

In total for both hospitals, the pathological examinations supported by clinical criteria allowed to diagnose 63 cases of gingival erosive lichens planus including 4 bullous lichens planus (mean age 60.9 years, 51 females, 12 males); 33 cases of autoimmune bullous diseases with gingival expression including 6 pemphigus vulgaris (mean age 46 years, 5 females, 1 male), 25 mucous membrane pemphigoids, and 2 pemphigoid lichen planus (mean age 66 years, 14 females, 13 males); 3 cases of plasma cell gingivitis (mean age 57 years, 2 females, 1 male); and 1 case of severe gingivitis only induced by dental plaque (1 male 71 years old) (Fig. 2 and Additional file 2). For the 33 patients with autoimmune bullous diseases, the additional examinations performed by the referring dermatologists (electronic microscopy, indirect immunofluorescence, immunotransfer and/or ELISA) allowed to confirm the initial diagnoses.

For the overall 100 patients, post-operative complications were almost non-existent. No haemorrhagic complications (gingival haemorrhage ≥ to 24 h), nor ecchymosis were observed during the postoperative phase. Only 4 patients reported a moderate transient bleeding within the hour following biopsy: 2 patients medicated with antihemorrhagic drugs, and 2 patients who did not follow the prescribed recommandations (maintaining a compress on the biopsy site during several minutes postoperatively, spitting forbidde). In the same way, no patient used pain medication following biopsy, gingival sensitivities varied from 0 to 2 according to a digital pain assessment scale scored 0–10. Wound healing always revealed satisfactory, with a clinical *ad integrum* papilla regeneration within 21 days following surgery (Fig. 1c). Long-term follow-up patients did not revealed loss of substance or postoperative scar in any patients.

## Discussion

Our two-center retrospective study including 148 tissue specimens allowed us to confirm that the interdental papilla biopsy technique is reproducible and highly efficient. Our surgical technique permitted us to establish the certainty diagnosis for 96 out of 100 patients. Only 5 biopsies had to be redone for 4 patients with a mucous membrane pemphigoid in a context of generalized and severe erosive gingivitis. The contribution level in both hospital departments were finally 94.7% for the Bretonneau Hospital and 97.1% for the Henri Mondor Hospital. Even though biopsy analyses were performed by different anatomopathologists, which could be a bias, histological examination with hematoxylin eosin and DIF are routine, perfectly codified and standardized examinations. The evaluation criteria are also well defined and known to hospital anatomopathologists [1, 4, 16, 17]. So, we believe that this approach does not change our results although bias is always possible. We have chosen to establish the level of contribution of the gingival specimens to diagnosis instead of taking into account the specificity and sensitivity calculations of the biopsy, as it is done in some studies [17, 20]. Indeed, the sensitivity and specificity of a diagnostic test allows to determine its ability to identify respectively sick people (true positives), and not sick people (true negatives). Regarding direct immunofluorescence, there cannot be false positives. The in situ positive autoantibody labelling confirms unmistakably that the patient has autoimmune bullous diseases, as the immunofluorence technique is nowadays very efficient. Moreover, in the autoimmune bullous diseases context, a non labelling can be obtained if the biopsy was performed distant from the site where the autoantibodies are located, i.e. where inflammation has destroyed them, or if the epithelium is completly cleaved from the connective tissue at the time of biopsy. In these cases, the result is interpreted as a false negative, while it must be related only to the surgical procedure. It is therefore inappropriate to talk about false positives and false negatives in the strict meaning of the term. On the other hand, it is possible to determine the level of contribution of the biopsy protocols to diagnosis by allowing a good quality of anatomopathological interpretation.

According to our experience, biopsy performed directly within the tissue targeted by the autoantibodies reduces the risk of not obtaining immunolabelling. To do this, you just have to perform the biopsy distant to the gingival erosive zones or the particularly inflammed areas. Our surgical protocol is also simple to perform by dental clinicians who are used to handling gingiva and presents a good tolerance regardless of the pathology causing chronic erosive gingivitis condition. The amount of available interdental gingival tissue is always sufficient even in the presence of periodontal pockets or intially reduced gingiva. Incisions can be sharp and clear from the start up to the bone contact because the gesture is guided by the tooth surface. Furthermore, the apical limit of the biopsy is always located within the attached gingiva and on the cortical bone. No sutures are required. Hemostasis is easily obtained by simple haemostatic compression. For patients with bleeding risk, a collagen or nitrocellulose pad may be placed directly on the biopsy site and replaced by the patient if necessary. The use of the punch technique for biopsy, which only allows to perform contour incisions, is not needed; it is the same for the use of tissue tweezers, which simplifies the surgical procedure while avoiding the risk of specimen damage by crushing it. In addition, healing of the papilla is rapid and *ad integrum* in less than 21 days postsurgery, with no risk of periodontal recession, because the entire buccal gingiva is preserved as its epithelio-connective attachement as well. Healing occurs starting from the gingival borders and particularly from the lingual intact papilla. Therefore, our biopsy protocol is also possible in the esthetic zones. The patient can eat and brush his teeth on the biopsy site from 3 to 4 days postsurgery.

**Fig. 3:**
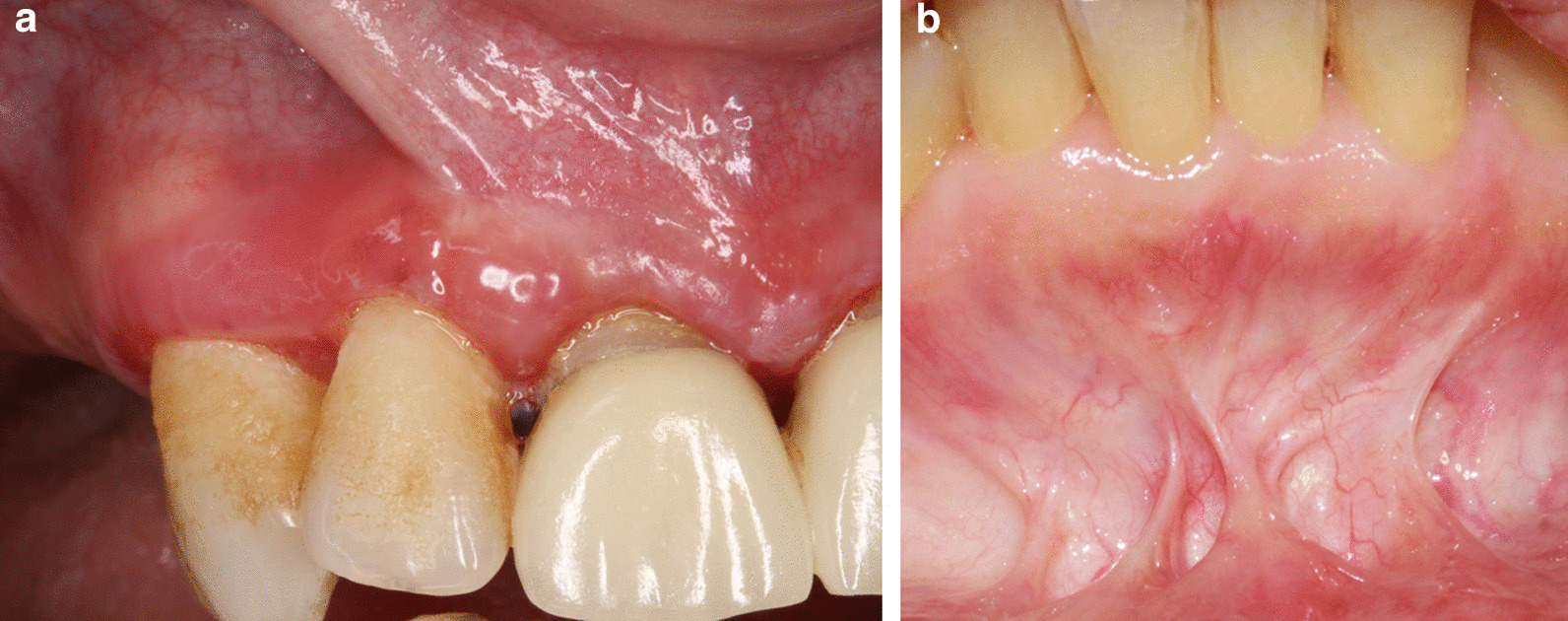
Examples of scar tissue that appeared after a biopsy on the alveolar mucosa in two patients with mucous membrane pemphigoid (**a**, **b**)

Other advantages may be of interest. The buccal papilla sites are numerous and accessible. If a haemorrhagic vesicle develops at the time of biopsy, it is immediately visible because the bleeding causes epithelial cleavage. Then you just have to change site with no healing inconvenience by staying in the anesthetized area. The surgery acte may be performed without any operating aid because the bleeding will be absorbed by sterile compresses positioned on the borders of the biopsy site. Avoiding surgical suction clearly limits the risk of epithelial cleavage. There is almost no post operative pain, and even in case of mucous membrane pemphigoid, no scar tissue can be observed at long term. Of course these lesions are not serious, but they can lead to tissue tension which are sources of discomfort and sensitivity for the patient (Fig. 3). To our knowledge, we are the only authors who have considered this type of long-term complications.

Our surgical procedure is close to the protocol proposed by Endo et al. in 2014 [17] and taken over by Gilvetti et al. in 2019 [20]. The first authors recommend to perform the biopsies within the attached gingiva, without harvesting any marginal gingiva, at the periphery of gingival lesions for standard histological examination, and distant from the lesions for direct immunofluorescence. Furthermore, they specify the interest of sharp incisions perpendicular to the cortical bone, up to bone contact, in order to avoid shear forces likely to cleave the epithelium. They also emphasize the need for gentle dissection by tilting the n°15 blade, in a second step, parallel to the bone surface (Stab-and-Roll technique). According to their protocole, the authors were able to obtain 51 contributive gingival biopsies out of the 52 performed except for 1 direct immunofluorescence out of 25 which could not be interpreted due to the absence of epithelium in the specimen, Gilvetti et al. [20] also present satisfactory and predictive results when the biopsies are performed within the attached gingiva (61 biopsies) compared to those performed on other oral sites: oral mucosa, palate, lip, toungue (64 biopsies). In their study, 72% and 34.37% of the biopsies were performed using a punch technique on the keratinized gingiva and on the other oral sites, respectively; 38% of patients had an autoimmune bullous diseases and 36% a lichen planus or a lichenoid lesion. The authors did not obtain contributory results for all sites and techniques for 14 out of 66 patients for routine histology, and 3 out of 45 patients for DIF. Furthermore, the authors note a better diagnostic performance when the biopsy is performed using the punch technique compared to the use of a conventional scalpel. However, although clinically valuable, this last surgical procedure has some disadvantages. If the disease causing erosive gingivitis occurs on an underlying periodontitis situation, the amount of attached gingiva can be reduced, which hence limits the extent of the harvested tissue specimen. Moreover, the apical limit of the incision risks to adjoin the mucogingival junction, which may lead to profuse bleeding in such circumstances. In this regard, Endo et al. [17] failed to specify whether they needed to suture the wounds, and provided no comment on the post-operative consequences. On the other hand, Gilvetti et al. [20] mention that they had to resort using sutures or bipolar diathermy to ensure haemostasis, but they point out in parallel the absence of short-term postoperative complications.

Otherwise, some clinicians contraindicate gingival biopsy by highlighting the fragility of inflammed gingival tissue. This is the case for Sano et al. [12] who include several oral biopsy sites in their study. However, the number of theis oral mucosa specimens are unequal, and their results non significant. So, it is difficult for them to demonstrate the superiority of one site over another. In addition, they provide no details on the protocol used for biopsy. Most recently, Carey et al. [19] also favour sampling sites within the alveolar mucosa located next to the gingival erosive lesions instead of the gingival areas. In mucous membrane pemphigoid cases, and using the punch technique, they obtain a positive direct immunofluorescence level which is significantly more favourable for the alveolar sites: 100% (17 biopsies /17) versus 84% (63 biopsies/75) for the gingival sites. However, the authors performed biopsy only within the attached gingiva without specifying either the surgical technique or the long-term quality of the soft tissue healing.

Other authors advise sampling only the gingival epithelium after inducing its detachment with a finger or instrument[13, 21]. However, this biopsy protocol is not feasible in case of pemphigus vulgaris because the epithelium cannot be detached. In case of erosive lichen planus because it does not allow for a complete histological analysis since the chorion is not removed.

## Conclusion

The interdental papilla biospy technique is ideal for the pathological examinations required to diagnose diseases for which the clinical translation is chronic erosive gingivitis. Two simultaneous biopsies, one essential for a standard histological examination, and the other for direct immunofluorescence, are recommended in case of atypical, severe, old chronic erosive gingivitis or chronic erosive gingivitis associated with blisters or pseudomembranes. This procedure is simple to perform, accessible to all clinicians with a high efficiency, an excellent contribution level and a good tolerance.

The clinical impact is real because the involvement of general clinicians (dentists, dermatologists) in detecting the diseases causing this type of non plaque-induced gingival pathologies is essential to reduce diagnostic delays and to send patients early to a referral hospital department. However, further research is needed to compare reproducibility rates between different biopsy protocols for chronic erosive gingivitis.

## Supplementary Information

**Additional file 1.** Performing a papillary biopsy in a patient with mucous membrane pemphigoid (video)

**Additional file 2. **Recapitulative table mentioning the pathologies the patient is suffering from, the patient characteristics and the number of cases of localized or generalized erosive gingivitis depending on the hospital where the patient has been taken care of. *LPG* gingival lichen planus, *AIBD* autoimmune bullous diseases, *PV* vulgar pemphigus, *MMP* mucous membrane pemphigoid, *PG* plasma cell gingivitis, *PIG* plaque induced gingivitis, *LEG* localized erosive gingivitis, *GEG* generalized erosive gingivitis, *F* female, *M* male

## Data Availability

Not applicable.

## Abbreviations

AIBD: autoimmune bullous diseases
PV: pemphigus vulgaris
MMP: mucous membrane pemphigoid
ABE: acquired bullous epidermolysis
LIgAD: linear immunoglobulin A dermatosis
PLP: pemphigoid lichen planus
DIF: direct immunofluorescence
HE: hematoxylin eosin
