# Supplementary material for: Clinical relevance of interdental papilla biopsy in chronic erosive gingivitis (desquamative gingivitis): retrospective bicentric study of 148 specimens
Source: BMC Oral Health. 2021 Sep 17;21:452. doi: 10.1186/s12903-021-01820-9 (PMC8447615; doi:10.1186/s12903-021-01820-9)
Supplement: Supplementary file 2 — Additional file 2. Recapitulative table mentioning the pathologies the patient is suffering from, the patient characteristics and the number of cases of localized or generalized erosive gingivitis depending on the hospital where the patient has been taken care of. LPG gingival lichen planus, AIBD autoimmune bullous diseases, PV vulgar pemphigus, MMP mucous membrane pemphigoid, PG plasma cell gingivitis, PIG plaque induced gingivitis, LEG localized erosive gingivitis, GEG generalized erosive gingivitis, F female, M male [file 12903_2021_1820_MOESM2_ESM.docx]

**Clinical relevance of interdental papilla biopsy in chronic erosive gingivitis (desquamative gingivitis) :**

**Retrospective bicentric study of 148 specimens**

**Authors**

Frédérick Gaultier^1^, Anne-laure Ejeil^2^, Sébastien Jungo^2^, Saskia Ingen-Housz-Oro^3,4,5^, François Le Pelletier de Clatigny^6^, Gogly Bruno ^1, 7^, Philippe Pirnay ^1^, Fadel Bellakhdar^1^, Sophie-Myriam Dridi^8,9^

1. Department of Odontology, Henri Mondor Hospital, Créteil, Université de Paris France
2. Department of Odontology, Bretonneau Hospital, Université de Paris France
3. Department of Dermatology, Henri Mondor Hospital, Créteil, France
4. Competence Centre of Autoimmune Bullous Diseases MALIBUL, FIMARAD Sector, Créteil, France
5. EA7379 EpidermE, UPEC, Créteil, France
6. Department of Pathology, Pitié Salpetrière Hospital, Paris France
7. Laboratory of Molecular Oral Pathophysiology, INSERM 1138, Université de Paris France.
8. Department of Odontology, Saint Roch Hospital, Nice France
9. Oral Microbiology, Immunotherapy and Health EA 7354, Nice, France

Additional file 2: Recapitulative table mentioning the pathologies the patient is suffering from, the patient characteristics and the number of cases of localized or generalized erosive gingivitis depending on the hospital where the patient has been taken care of.

| Pathologies  (Type, Number, %, Hospital) | Patient characteristics  (Age (SD), gender) | Type of erosive gingivitis |
| --- | --- | --- |
| n = 100 (100%)  n (Mondor) = 75  n (Bretonneau) = 25 | **A(m) = 61,4 (23 - 93) - 72 F/28 M** | **43 LEG – 57 GEG** |
| GLP n = 63 ; 63%  Bretonneau Hospital (n= 17)  Typical GLP n = 13  Related GLP n = 2  Bullous GLP n = 2  Henri Mondor Hospital (n = 46)  Typical GLP n = 22  Related GLP n = 22  Bullous GLP n= 2 | **60,9 (37-93) - 51 F/12 M**  60 (37-75) - 11 F/2 M  67,5 (64-71) - 1 F/1 M  66 (62-70) - 2F  59,2 (35-81) - 19 F/3 M  62,4 (23-93) - 16 F/ 6 M  57,5 (44-71) - 2 F | **32 LEG, 31 GEG**  5 LEG, 8 GEG  2 GEG  2 GEG  14 LEG, 8 GEG  14 LEG, 8 GEG  2 GEG |
| AIBD n = 33 ; 33%  Bretonneau Hospital (n = 8)  PV n = 3  MMP n = 5  Henri Mondor Hospital (n = 25)  PV n= 3  MMP n = 22 | **62,41 (23-87) - 19 F/14 M**  62 (33-84) - 3 F  62,8 (49-77) - 2 F/3 M  30 (23-35) - 2 F/1 M  66,8 (56-87) - 12 F/10 M | **7 LEG, 26 GEG**  3 GEG  2 LEG, 3 GEG  3 GEG  5 LEG, 17 GEG |
| PG (n = 3); 3%  Henri Mondor Hospital | **57,6 (37-78) - 2 F/1 M** | **3 LEG** |
| PIG (n = 1) ; 1%  Bretonneau Hospital | **71 - M** | **LEG** |
